# Immunotoxins: The Role of the Toxin ^†^

**DOI:** 10.3390/toxins5081486

**Published:** 2013-08-21

**Authors:** Antonella Antignani, David FitzGerald

**Affiliations:** Biotherapy Section, Laboratory of Molecular Biology, Center for Cancer Research, National Cancer Institute, 37 Convent Dr, Bethesda, MD 20892, USA

**Keywords:** immunotoxin, antibody, toxin, cancer, immunotherapy, apoptosis, translocation, ricin, diphtheria, *Pseudomonas*

## Abstract

Immunotoxins are antibody-toxin bifunctional molecules that rely on intracellular toxin action to kill target cells. Target specificity is determined via the binding attributes of the chosen antibody. Mostly, but not exclusively, immunotoxins are purpose-built to kill cancer cells as part of novel treatment approaches. Other applications for immunotoxins include immune regulation and the treatment of viral or parasitic diseases. Here we discuss the utility of protein toxins, of both bacterial and plant origin, joined to antibodies for targeting cancer cells. Finally, while clinical goals are focused on the development of novel cancer treatments, much has been learned about toxin action and intracellular pathways. Thus toxins are considered both medicines for treating human disease and probes of cellular function.

## 1. Introduction

In the late 1970s three seminal papers set the stage for future immunotoxin development. One by Yamaizumi *et al.* confirmed the potency of diphtheria toxin for mammalian cells [1] and coined the now famous phrase “one molecule of diphtheria toxin (DT) can kill a cell”. Thus the potency of DT and similar protein toxins was established. Potency apparently resides in both the turnover rate and intracellular stability of the toxin’s enzyme domain. The second paper, by Thorpe *et al*., introduced the concept of using antibodies to redirect toxin killing activity in a purposeful way [2]. Specifically, the report described the use of anti-lymphocyte antibodies to kill lymphoblastoid tumor cells. The strategy involved the use of chemical linking agents to attach DT to these antibodies and so “early” immunotoxins were born. And, finally, the “antibody world” itself changed as monoclonal antibodies emerged onto the scene [3] allowing for the construction of bimolecular agents with toxins chemically attached to antibodies of a single defined specificity [4]. Then for a while “favorite” monoclonal antibodies were attached chemically to “favorite” toxins and new agents were produced on a regular basis, mostly for cancer therapy [5,6,7,8,9]. The next leap forward involved the application of molecular cloning techniques. This allowed for the production of fusion proteins composed of antibody fragments joined to enzymatically active toxin domains [10,11]. Mostly these fusion proteins were expressed in *E. coli*, which allowed for efficient production of a homogeneous product. 

Over 30 years of development, progress with immunotoxins as cancer treatment agents followed a predictable path: promising results in tissue culture systems led to experimentation in animal tumor models which progressed to large animal toxicology/pharmacology studies and then to the planning and implementation of clinical trials. Various immunotoxins derived either from the plant toxin ricin or the bacterial toxins DT or *Pseudomonas* exotoxin (PE) entered clinical trials. Many of these trials are now published with some describing very encouraging results [12,13,14,15,16,17,18,19,20,21,22,23,24]. However, also described are dose-limiting toxicities: including vascular leak syndrome, hemolytic uremic syndrome and pluritis [21,25,26]. Improved immunotoxin design should minimize these side effects. To date only one targeted toxin, DT-IL2 (termed denileukin diftitox—trade name Ontak), directed to the IL2 receptor, has been approved for human use [27,28]. The approval of other immunotoxins awaits favorable results from Phase III trials. Despite having a reputation for potency, immunotoxins have been co-administered with “enhancing agents” even from the earliest days—in the hopes of making a good reagent even better [29,30,31]. Because cancer therapies usually require combination treatments this is not an unreasonable approach: and, in the future, successful immunotoxin development will likely depend on discovering the best agents for co-administration. 

While immunotoxins are most frequently studied as cancer therapy agents other uses have been suggested and evaluated—for a recent comprehensive immunotoxin review see Shapira and Benhar [32]. These include modulating immune responses: such as preventing graft versus host disease [33,34], removing T-cells from grafts [35,36] or the elimination T-regulatory cells [37,38,39,40]. Some progress has been made also in producing immunotoxins with anti-viral [41,42,43] or anti-parasitic activity [44]. *Ex-vivo* uses are also anticipated whereby unwanted cells are killed before infusing bone marrow or other stem cell like preparations [45,46]. 

Immunotoxin experimentation with eukaryotic cells has led directly to the identification of novel toxin features and functional domains. Similarly, the concept of toxins-as-probes of eukaryotic biology has been exploited to uncover previously unknown pathways or properties of cells. A very early example of the latter stemmed from the observation in the 1960s by Kim and Groman that ammonium chloride protected cells from DT [47]. This led to the understanding that endocytic vesicles are maintained at acidic pH. And as we now know, acidic pH is required for DT transport to the cytosol [48,49].

## 2. Toxin Candidates

Protein toxins first came to prominence as pathogenic factors released by bacteria or poisons ingested from toxic plants and were noteworthy because, as “single agents”, they caused such severe morbidity and mortality. Many decades later, it is intriguing to note that several of these toxins share a common biochemical mechanism *i*.*e*., they inhibit protein synthesis. DT and PE have similar mechanisms: they ADP-ribosylate elongation factor 2 and halt protein synthesis at the elongation step [50]. Ricin A chain is an *N*-glycosidase and is toxic because it depurinates a critical adenine in 28S rRNA [51]. And it is from these toxins that investigators have turned most often to construct immunotoxins. Originally there were three toxin candidates: the plant toxin ricin (and similar toxins expressed from other plants [52,53]) and the bacterial toxins DT and PE. Because of toxin complexity and reagent loyalty, rarely did individual researchers use more than one toxin, so direct toxin-to-toxin comparisons were seldom undertaken. Even to this day, these three toxins remain among the top choices for immunotoxin development; although, other plant toxins and fungal toxins are also used to make immunotoxins [53,54]. So what were the features that characterize toxin utility?

**Figure 1:**
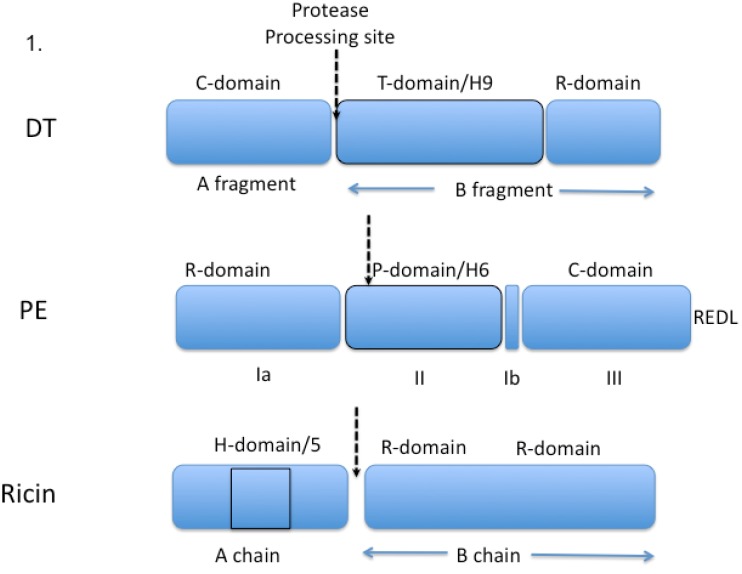
Graphic representations of three toxins, diphtheria toxin (DT), *Pseudomonas* exotoxin (PE) and the plant toxin, ricin. Above each “domain” is a functional label. Below each domain is a common name that was used in early publications. DT has an *N*-terminus catalytic domain (C-domain) also known and the A fragment followed by a protease processing site, then a nine helix domain (commonly known as the “T” or translocation-domain) followed by a receptor binding domain (R-domain). The B-fragment includes both the T-domain and the R-domain. PE has an *N*-terminal receptor-binding domain followed by a processing domain. Then at the *C*-terminus there is a catalytic domain followed by a KDEL-like sequence. Ricin has a catalytic domain at the *N*-terminus, followed by a processing site and then a duplicated receptor-binding domain with a preference for binding galactose residues. Each toxin has a helical domain where several helices follow in close sequence. For DT there are nine helices while PE has 6; and these helices are arranged in what appears to be a separate domain between C and R-domains. Ricin also has a cluster of helices but these are located in the middle of its catalytic domain. A simple view of these helical domains is that they function in the translocation of each toxin’s C-domain. However, this has only been established for the T-domain of DT. The site of proteolytic processing is shown for each toxin.

Toxin structure, orientation of domains, expression and purification yields, ease of cloning, sugar binding, immunogenicity, and non-specific toxicity have each contributed to researchers choosing to work with one toxin over another. Many of these issues have been discussed in a recent review [32] and won’t be discussed at length here but a few key points should be mentioned. Each toxin has an active enzyme domain that must reach the cell cytosol to kill cells. Each toxin also has a cell-binding domain that has to be eliminated or nullified before attachment to an antibody. Finally there is the “translocation” function, which may or may not be encompassed in a single functional domain. The “job” of the translocation domain is the transport of the toxin’s enzyme domain across an intracellular membrane into the cell cytosol. And, even today, understanding the mechanism or mechanisms of toxin translocation remains a challenge. In broad terms, DT translocates from acidic endosomes with the aid of its T-domain [55], while ricin and PE associate with the ER prior to translocation; although the case for the ER pathway is stronger for PE [56] (with a known KDEL-like sequence at the *C*-terminus) than it is for ricin [57,58]. For each toxin, translocation apparently involves unfolding prior to reaching the cytosol [59,60] and refolding once in the cytosol, leading the speculation that chaperones may be needed for the most efficient translocation [61]. DT and PE have distinct binding and enzymatic domains-at each termini and an alpha helical domain in the middle (Figure 1). The role of the helical domain is more clearly defined for DT than for PE but it is intriguing to note that multi-helical domains of protein toxins may be involved in membrane insertion and possible pore formation [62]. In fact, the membrane insertion of the T domain of DT has been used to model the molecular behavior of Bax and Bak, the proapoptosis Bcl2 proteins that cause pores in mitochondria, leading to the release of cytochrome C and the initiation of apoptosis [62]. In the case of ricin distinct binding (the B chain) and enzyme domains (the A chain) are also defined (Figure 1) while translocation activity is harder to locate precisely. However, it is noteworthy to point out the presence of a 5-helix structure in the middle of the A chain. Ricin A (RTA) is clearly able to translocate to the cytosol when coupled to some monoclonal antibodies [63]. And several trials are on-going evaluating the utility of this form of the toxin [19,63]. However, when RTA is coupled to other antibodies, there is poor cell killing and researchers are “forced” to include the B chain as well [22,64] suggesting that in some instances the B chain is needed to direct the routing of the A chain. To nullify normal B chain binding to surface galactose residues, immunotoxins were developed using “blocked” ricin. Blocked ricin retains sugar-binding residues but their active sites are blocked via chemical modification. Retaining the entire B-chain, albeit with reduced binding activity, has also been reported for DT immunotoxins constructed with CRM9 [35,36].

Finally, toxins that interact with mammalian cells invariably need “processing” steps to convert a precursor molecule to an active one [65]. In addition, toxins act in the cell cytosol and must reach their destination via a collaboration between the toxin and the target cell [57]. By tracking the fate of toxin molecules one can learn about cellular functions and thus toxins are probes for the cells they attack. For DT and PE minimum processing includes a protease cleavage step [66] (Figure 1) and a reduction of a key disulfide bond [67]. Other features include a transient unfolding step-followed by refolding [61] For PE, there is a KDEL-like ER retention sequence at the *C*-terminus that is essential for cell killing activity [56]. So for PE and PE-derived immunotoxins there are four known steps prior to reaching the cytosol: (1) receptor binding, (2) furin cleavage, (3) disulfide reduction and (4) interaction with KDEL receptor 2. For DT in addition to protease “nicking” there is a cytosolic chaperone and reductase that have been identified as being important for toxin action [61]. Proteolytic processing of ricin occurs in the germinating castor bean, producing the A and B chains (Figure 1). Because ricin interacts with terminal galactose residues displayed on many different surface receptors, tracking its fate can be challenging [68,69]. Ricin also requires an intracellular reduction step. Recent studies using RNAi highlighted important genes in the ricin pathway and compared these with genes involved in PE intoxication [70,71]. These genetic screens along with chemical screens to identify anti-ricin compounds should provide new insights into ricin’s intracellular trafficking pathway [72].

## 3. Early Immunotoxin Development

Thorpe *et al.* set the stage for immunotoxin development by confirming that protein toxins could be redirected to kill selected cell types over bystander cells [2]. However, their result was achieved with a poorly defined antibody preparation. Using the same concept but with the benefit of Kohler and Milstein’s monoclonal antibody technology [3,73], well defined immunotoxins of a single specificity were produced. These included, ricin-, DT- and PE-derived immunotoxins. Besides antibody and toxin selection, other steps in the manufacture of immunotoxins included the use of different chemical “glues” (called cross linkers) to join the two molecules in a manner that kept both parts functional [74,75]. Early on it was appreciated that antibodies alone were rarely cytotoxic. This fueled research into making antibodies more potent by attaching protein toxins to them. Potency depended not only on internalization but also on the “correct” internal conditions within the cell. For instance, in the case of early immunotoxins to CD5 made with the T101 antibody, neutralization of acidic pH was deemed important for optimal killing [76]. In other immunotoxins, disulfide linkers allowed for cytotoxic activity while thioether linkers did not, confirming the need for the appropriate reducing environment to allow separation of toxin from antibody [75,77]. 

**Figure 2:**
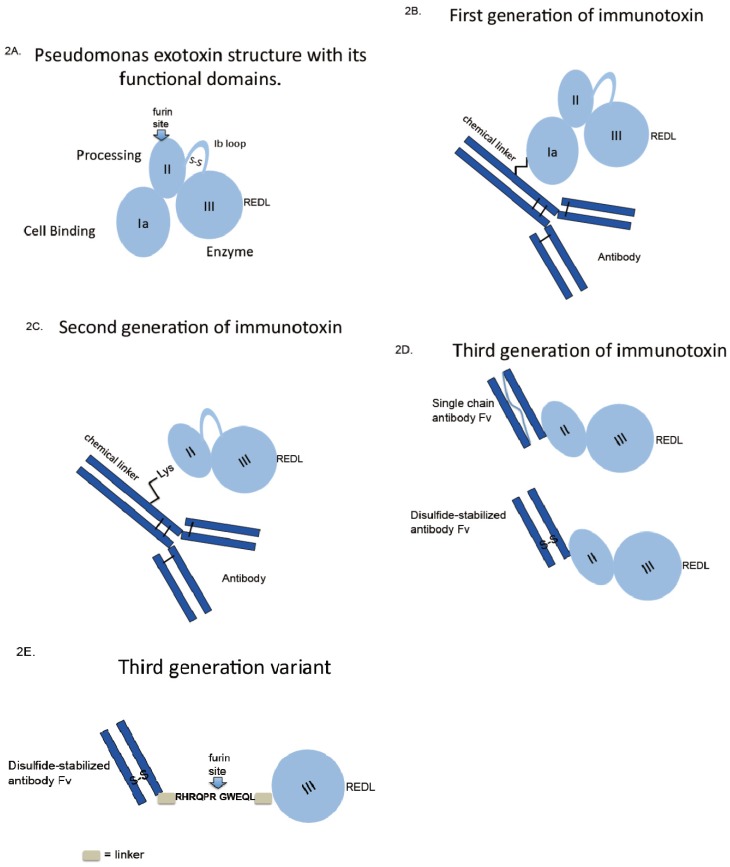
Immunotoxin construction-from oldest to newest. First generation immunotoxins were constructed by using chemical crosslinking agents to attach intact toxins to intact antibodies. Second generation immunotoxins used modified toxins lacking receptor-binding domains. Third generation molecules used cloned antibody fragments fused to modified toxin genes; allowing for the recombinant production of homogeneous protein. Further improvements of the third generation molecule might include the removal of immunogenic amino acids including (as shown) much of the multi-helical domain of PE.

For PE the first immunotoxins were made via thioether linkage from an intact monoclonal antibody to the native intact toxin (Figure 2B). When the functions of the toxin’s structural domain were discovered, it made sense to delete the receptor binding domain, producing a molecule termed PE40-based on its molecular weight. However, the deletion of the *N*-terminal domain (harboring many lysine residues for chemical conjugation) created a problem of how to attach PE40 to antibodies. This was solved by the introduction of a novel lysine residue near the terminus of PE40, producing Lys-PE40 (Figure 2C). Together, these chemical conjugates made up first and second generations of immunotoxins. 

## 4. Evolved Immunotoxins

Molecular cloning techniques, producing gene fusions, together with prokaryotic expression systems revolutionized the development of immunotoxins and has produced a third generation molecule (Figure 2D). No longer was it necessary to purify large quantities of antibody and toxin and then combine the two using chemical cross linkers. The latter approach was not very efficient, produced heterogeneous products and risked interfering with antigen binding. The initial breakthrough for recombinant immunotoxins came with the expression of single chain Fv fragments in *E. coli* that retained antigen binding [78,79]. This led directly to the first recombinant antibody-toxin fusion protein [80]. For PE-based immunotoxins this placed the antibody fragment at the *N*-terminus leaving the KDEL-like sequence free at the *C*-terminus (Figure 2D). While this approach was very attractive for the design of recombinant cytotoxic molecules, several challenges remained for expression and purification of active monomeric species. Codon optimization (mostly avoiding rare Arg codons in the antibody clone), inclusion body production, refolding protocols that included redox-shuffling and a multi-step purification scheme solved most of the problems [81,82]. One problem that was not immediately obvious related to the propensity of single chain antibody fragments to aggregate. For unstable Fvs, this issue was overcome with the incorporation of a novel disulfide linker that replaced the flexible linker originally described to keep the heavy and light chains tethered to one another [83,84]. Once produced, immunotoxins directed to potential cancer targets were investigated for cell killing activity and for non-specific damage to non-target tissue. Of various candidate antigens, mesothelin, CD22 and CD25 remain options for clinical investigation [85,86,87]. Other antigens including HER2/neu, Lewis-Y, CD30 and CD19 were considered for pre-clinical development but were either abandoned because of systemic toxicity or never developed because of poor cytotoxic activity in tissue culture.

More recent advances in the development of recombinant immunotoxins have focused on the production of smaller and less immunogenic versions of the original PE40/38 molecule [88,89,90]. By eliminating most of domain II of PE, a smaller molecule was produced that retained cytotoxic action with the added benefit of removal of one major and several minor immunogenic epitopes. The production of a smaller version of PE-derived immunotoxins was the by-product of an effort to stabilize the toxin intracellularly and prevent degradation in lysosomes. The deletion of domain II also removed many lysosomal cleavage sites and produced a molecule that was termed “LR” for lysosomal resistant [88]. Thus the LR version of PE-derived immunotoxins exhibits three new features: it is smaller, less immunogenic and more resistant to cleavage by lysosomal enzymes [89,90]. Generating molecules that lack domain II also produced a “puzzle”: these immunotoxins are more active against various cell types and less active against others [88]. A full explanation for this disparity remains to be uncovered as does a full appreciation of the role of domain II in PE-mediated killing. However, the removal of domain II allowed for the production of a “minimal” immunotoxin. This path of development was summarized in a recent review [91]. Data support the retention of an *N*-terminal antibody Fv, linked by a minimal furin site to domain III and the placement of a KDEL-like sequence at the *C*-terminus—see Figure 2E. 

## 5. Gene Fusions with Cell-Binding Ligands

Classically, immunotoxins describe an antibody-toxin molecule, either linked or fused together. However, receptor ligands can also be used to direct toxins to kill cells expressing specific target receptors. EGF was an early candidate in this class of ligand-toxin molecule followed by TGFalpha, IL-2, IL-4, IL-6, IL-13 and with diphtheria toxin, MSH, TF, and IL-2 [27,92,93,94]. Ligand-toxins can be efficient at killing cells displaying the corresponding receptor but potentially send a “mixed” message to cells. Many peptide ligands convey growth or survival signals via binding to surface receptors. These signals are often transmitted via phosphorylation cascades and happen quite rapidly. Thus the signal to grow or survive can be transmitted perhaps several hours before the toxin gains access to the cytosol and begins to shut down protein synthesis. Despite these concerns, it is noteworthy that the only “immunotoxin” approved for treatment to date is a DT-IL2 toxin termed, denileukin diftitox. 

## 6. Toxin Resistance

Three kinds of toxin resistance are known: cellular, organismal and immunologic. Cellular resistance is further divided into inefficient delivery to the cytosol [95,96], failure to ADPr EF2 [97] or poor triggering of apoptosis [98]. At the level of the intact animal or person, delivery to every cell can be challenging and may require either repeated dosing or dosing together with agents that improve access to tumor cells [99,100]. Finally, immunologic resistance arises when patients with intact immune systems make neutralizing antibodies to the immunotoxin (usually the toxin portion of the immunotoxin) [26,101]. For each type of resistance listed above, it is possible to make improvements in design of the immunotoxin itself that should produce better anti-tumor results. That said, it will be additionally useful to design treatment options that include co-administration of “helper” agents that overcome the particular resistance of concern—see below. 

## 7. Immunotoxin-Drug Combinations

Even from early days—combinations were sought to make immunotoxins more active. For example, ammonium chloride or monensin was added to cultured cells to make them more sensitive to ricin A immunotoxins [102,103]. Likewise calcium channel blockers enhanced the activity of PE-based immunotoxins [104]. Co-delivery with endosome-disrupting adenovirus also enhanced PE immunotoxins [105]. None of these approaches was ever likely to be useful *in vivo* because it would be difficult to achieve the necessary drug levels or overcome safety concerns. However, these results confirmed the utility of using combinations to increase the delivery of toxins to the cytosol and also highlighted a potential issue of inefficient trafficking of toxins within target cells. Expanding on this concept was a study by Youle *et al.* showing that retinoic acid disrupted Golgi structure and in the process enhanced the activity of a ricin A chain immunotoxin by 10,000-fold. Like earlier studies, the authors concluded that the enhanced killing was a result of increased delivery of toxin to the cytosol [106].

A second reason for using enhancers has been proposed more recently and that is to decrease the influence of prosurvival factors that might otherwise prevent cells from succumbing to cytotoxic therapy [107,108,109]. A similar strategy employs targeting agents to “death” receptors has also been explored as a way to overcome resistance to apoptosis [110]. 

At the level of the intact animal, there are several reports of using combination treatments *in vivo* to enhance immunotoxin-mediated antitumor action. Agents that enhance immunotoxin access to tumor cells such as taxol, produce improved responses over either agent alone [100,111]. The report from 2007 suggests that local concentrations of shed antigen were also reduced with taxol treatment, potentially allowing immunotoxin molecules less restricted access to tumor cells [100]. Similarly, agents that neutralize survival factors, such as Bcl-2 proteins, may enhance immunotoxin action. In several tumor systems this approach appears to have merit where the presence of the Bcl2/Bcl-xl inhibitor ABT-737 enhanced immunotoxin-mediated antitumor action [98,109].

If we examine on-going clinical trials, we see these kinds of efforts persist. Combotox was developed to improve the antitumor action of ricin A chain-based immunotoxins: two immunotoxins are administered in a 1:1 ratio. By combining an anti-CD22 immunotoxin with an anti-CD19 immunotoxin it is hoped to avoid resistance and to produce improved treatment outcomes compared to the use of either agent alone [20,21]. Phase 1 studies’ results with this combination approach produced encouraging results and a recent trial was opened using Combotox in conjunction with cytarabine for patients with B-cell ALL (ClinicalTrials.gov Identifier: NCT01408160). Likewise, in an effort to enhance patient responses to the SS1P immunotoxin targeting surface mesothelin, a current trial investigates the co-administration of the immunosuppressive agents cytoxan and pentastatin (ClinicalTrials.gov Identifier: NCT01362790).

## 8. Future Directions

Combinations to improve killing kinetics of immunotoxins or those that prevent the expression of survival pathways are likely to be particularly useful. Likewise, agents that break up tumor masses and allow greater access of immunotoxin to individual malignant cells should improve outcomes. Finally, agents that suppress neutralizing antibodies and that can be administered without severe toxicity, should be explored. 

## 9. Conclusions

Immunotoxins exhibit selectivity and potency for cancer cells and may be effective clinically as single agents only under exceptional circumstances (such as the targeting of circulating tumor cells expressing a high number of target antigens). Under more usual circumstances, immunotoxins may be beneficial as part of a combined treatment with other agents. The process of discovering agents that work best in combination with immunotoxins is currently ongoing. When combinations are needed, agents that increase toxin killing and reduce immunogenicity will likely be the most valuable ones.
